# Long Non-coding RNA Signature for Liver Metastasis of Colorectal Cancers

**DOI:** 10.3389/fcell.2021.707115

**Published:** 2021-07-08

**Authors:** Fang Liu, Zhen-Mei Song, Xiao-Di Wang, Shi-Yu Du, Na Peng, Jing-Rui Zhou, Ming-Gang Zhang

**Affiliations:** ^1^Department of Gastroenterology, China-Japan Friendship Hospital, Beijing, China; ^2^Department of Gastroenterology, Shan GU Hospital, Handan, China

**Keywords:** colorectal cancer, liver metastasis, HOTAIR, MALAT1, LOC285194

## Abstract

Colorectal cancer ranks within the top three cancers both in terms of incidence as well as deaths. Metastasis is often the major cause of mortality and liver is the primary and most common site to which colorectal cancers metastasize. We tested the prognostic ability of a long non-coding RNA (lncRNA) signature in liver metastatic colorectal cancers. We first evaluated expression levels of several lncRNAs in eight excised liver metastases from primary colorectal cancers and found significantly upregulated lncRNAs HOTAIR and MALAT1 along with significantly downregulated LOC285194. We further compared the expression levels of HOTAIR, MALAT1 and LOC285194 in primary colorectal tumors at the time of initial diagnosis and correlated them with disease progression and liver metastasis. HOTAIR and MALAT1 were significantly upregulated and LOC285194 was significantly downregulated in twelve patients who were diagnosed with liver metastasis within 5 years of initial diagnosis, compared to the five patients with no metastasis. A positive signature comprising of high HOTAIR/MALAT1 and low LOC285194 also correlated with progression to higher grade tumors. Thus, the lncRNA signature comprising of high HOTAIR/MALAT1 and low LOC285194 could be a prognostic signature for liver metastasis as well as overall poor survival.

## Introduction

Colorectal cancers are some of the most frequently diagnosed cancers worldwide (Ferlay et al., 2021). In the United States, colorectal cancers are the third most commonly diagnosed cancers as well as the third most common cause of cancer-related deaths, in both males as well as females (Siegel et al., 2021). More than half of the patients diagnosed with this cancer succumb to this disease (Riihimaki et al., 2012). China accounts for almost 30% of global cancer deaths with colorectal cancers being rated among the top five and the deaths dues to liver cancer increasing in the numbers (Cao et al., 2021). Like almost all other human cancers, colorectal cancers can metastasize and such metastatic colorectal cancers are the primary cause of death. To make matters worse, it is believed that approximately one in five patients diagnosed with colorectal cancers already have metastatic disease at the time of diagnosis (van der Geest et al., 2015) and almost one in four patients have metastatic disease at the time of primary resection (Valderrama-Trevino et al., 2017). While there is evidence that the overall survival is better in patients with solitary lung or liver metastases (Riihimaki et al., 2016), the overall mortality due to metastatic diseases remains an issue of concern.

Liver is the primary site of metastasis in patients with metastatic colorectal cancer (Valderrama-Trevino et al., 2017; Zarour et al., 2017). As many as 70% of patients diagnosed with primary colorectal cancer are expected to develop liver metastases (Valderrama-Trevino et al., 2017). Interestingly, it has been reported that there are differences in the survival rates of right-sided vs. left-sided liver metastatic colorectal cancers with significantly higher mortality rates associated with the right-sided liver metastatic cancers (Engstrand et al., 2018). For the unresectable liver metastases due to the large size of metastases or the involvement of multiple nodes, it has been reported that treating patients with a combination of 5-fluorouracil, leucovorin and oxaliplatin can reduce tumor size making them presentable for surgery and resulting in significantly increased survival (Giacchetti et al., 1999). Liver metastases are just not more prevalent, but the associated mortality rate is much higher as well, compared to, for example, patients with lung metastases (Wang et al., 2020).

The power of non-coding RNAs as colorectal cancer prognostic biomarkers is undeniable (Zarate et al., 2012; Ferracin et al., 2016; Ahadi, 2020). In recent years, such power of a subtype of non-coding RNAs, the long non-coding RNAs (lncRNAs) has been reported by several investigators (Chen et al., 2014; Han et al., 2015; Li et al., 2016a) with indications for the ability of lncRNAs to play a role in liver metastases of colorectal cancers (Ye et al., 2015). We designed this study to first elucidate the lncRNAs that are differentially expressed in liver metastatic colorectal cancers and then to correlate an lncRNA-based signature with disease progression and outcome.

## Materials and Methods

### Patients

All patients were enrolled at China-Japan Friendship Hospital. The study was conducted after approval from the Ethics Committee at the China-Japan Friendship Hospital (Approval Number 5647). Informed consent was obtained from all patients prior to the collection of samples. For the initial assessment of lncRNAs, eight patients’ samples were analyzed (Table 1). These samples were selected based on liver metastasis diagnosis and the availability of adjacent non-cancer liver tissues. For the validation of lncRNA signature, 17 patients’ samples were analyzed. Only those samples were evaluated that had complete medical history so that the expression levels could be correlated with progression of disease and the disease outcome. Exclusion criteria was the diagnosis of metastatic disease at initial cancer diagnosis.

**TABLE 1:** Patient data.

**Gender**	**Number (Total = 8)**	**Liver metastases**	**Other metastases**
Males	5 (62.5%)	5 (100%)	1 (20%)
Females	3 (37.5%)	3 (100%)	0 (0%)

### lncRNA Detection

Primers and detection reagents were purchased from Qiagen (China) to detect lncRNAs in samples. Only RNAse-free water was used throughout the assays. RT^2^ First Strand Kit (Qiagen, China) was used for the synthesis of cDNA to eliminate genomic DNA. The starting amount of RNA was 1 μg to which 2 μl of genomic DNA elimination mix was added and mixed by pipetting, followed by incubation for 5 min at 42°C and then immediate transfer to ice for 1 min. Reverse transcription mix, consisting of 5× buffer and Reverse Transcriptase, was prepared exactly as suggested and added to the tube containing RNA. This was incubated for 15 min at 42°C and then the reaction stopped by transfer for 5 min to 95°C.

RT^2^ lncRNA qPCR assay (Qiagen, China) was used for the detection of lncRNAs. The product from RT^2^ First strand step was mixed with RT^2^ SYBR green mastermix (Qiagen, China) and run on an ABI 7500 RT-PCR system (Applied Biosystems) with the following PCR cycle conditions—1 cycle—10 min/95°C, followed by 40 cycles consisting of two steps—15 s/95°C and 1 min/60°C.

### Statistics

All data was evaluated by a biostatistician, who was blinded to the identity of individual patients. To evaluate if two datasets were significantly different, a *p* value was calculated using Student *t* test or one way ANOVA assuming equal variables and 2-tailed distribution. Prior to the statistical tests, datasets were log-transformed to ensure normal distribution. Only the *p* values ≤ 0.05 were considered to represent statistically significant analyses.

## Results

### Upregulated lncRNAs in Liver Metastatic Colorectal Cancers

**FIGURE 1:**
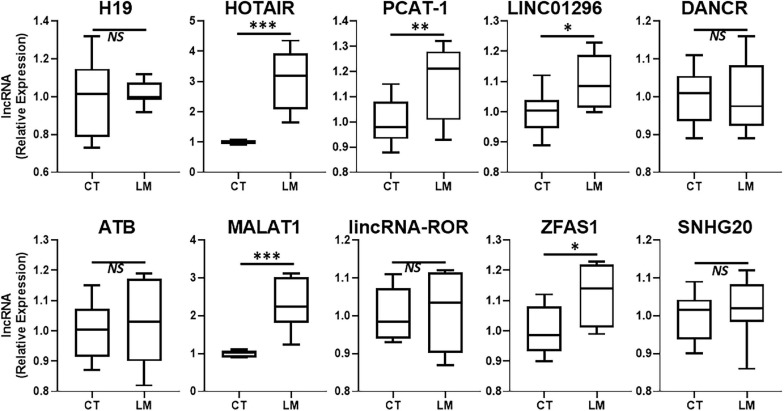
Upregulated lncRNAs in colorectal cancer liver metastases. Expression levels of several lncRNAs were tested in liver metastases of primary colorectal cancers (LM), by qRT-PCR, and compared to their expression levels in adjacent non-cancer liver tissues (CT: control). *N* = 8 *p < 0.05, **p < 0.01, ***p < 0.001, and *NS*, Non-significant.

Our focus for this study was to evaluate a role of lncRNAs in liver metastases of primary colorectal cancers. A number of lncRNAs have been proposed to influence colorectal cancer metastases and additionally, a number of lncRNAs can influence metastases of other primary cancers as well. We detected the expression levels of several such lncRNAs in the excised liver metastases of primary colorectal cancers and compared the expression levels of those lncRNAs in the adjacent non-cancerous liver tissue. We reasoned that this approach would provide a good indication of differentially expressed lncRNAs which could be associated with liver metastatic colorectal cancers. Since some lncRNAs are upregulated in metastatic disease, we first evaluated several such lncRNAs and the results are presented in Figure 1. The two lncRNAs that stood out as the most significantly upregulated (*p* < 0.0001) in liver metastases were the lncRNAs HOTAIR and MALAT1. PCAT-1 was significantly expressed with *p* < 0.01 while lncRNAs LINC01296 and ZFAS1 were differentially expressed with *p* < 0.05. HOTAIR and MALAT1 were the two lncRNAs of which the expression in liver metastases was always observed to be higher than the adjacent non-cancer tissues.

### Downregulated lncRNAs in Liver Metastatic Colorectal Cancers

**FIGURE 2:**
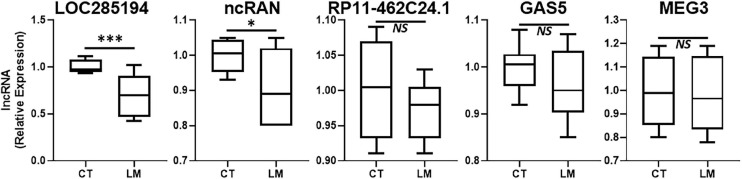
Downregulated lncRNAs in colorectal cancer liver metastases. Expression levels of several lncRNAs were tested in liver metastases (LM) of primary colorectal cancers, by qRT-PCR, and compared to their expression levels in adjacent non-cancer liver tissues (CT: control). *N* = 8 *p < 0.05, ***p < 0.001, and *NS*, Non-significant.

In addition to lncRNAs that are upregulated during disease progression, there are several lncRNAs that are actually downregulated. Such lncRNAs also need to be identified for possible therapy. We evaluated several such lncRNAs that are reported in literature to be “tumor-suppressive.” The results from our evaluations are presented in Figure 2. LOC285194 stood out as the more consistently downregulated lncRNA in liver metastases when compared to its expression in surrounding normal liver tissue. lncRNA ncRAN was also significantly differentially expressed but its level of significance (*p* < 0.05) was relatively less when compared to LOC285194 (*p* < 0.001).

### lncRNA Signature and Its Association With Liver Metastasis

**FIGURE 3:**
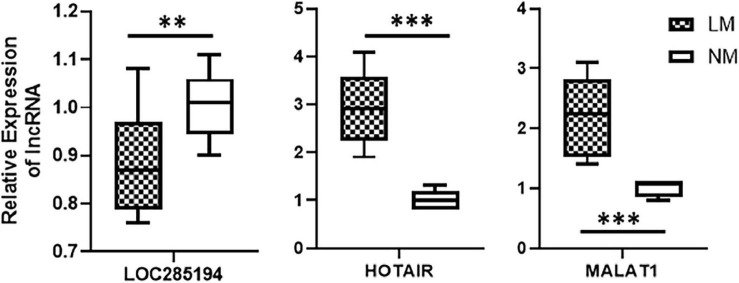
Association of lncRNAs with liver metastases. Expression levels of lncRNAs were tested in primary colorectal cancers, by qRT-PCR, collected at the time of initial diagnosis. Expression levels in *n* = 12 patients who reported liver metastases (LM) within 5 years of diagnosis were compared with expression levels in *n* = 5 patients with no reported metastases (NM: no metastasis). **p < 0.01, ***p < 0.001.

After the identification of a lncRNA signature comprising of three highly differentially expressed lncRNAs viz. HOTAIR, MALAT1, and LOC285194, we tested whether this lncRNA signature can actually be associated with liver metastases. We obtained patient samples (primary colorectal cancer tissues) that were excised from patients at the time of their initial diagnosis. Our rationale was to check if the lncRNA could be associated with liver metastases. We obtained a total of 17 samples with 12 samples from patients who were diagnosed with liver metastases within 5 years of initial diagnosis and the rest five samples from patients who were not diagnosed with any metastasis at least within 5 years of initial diagnosis. An evaluation of three individual lncRNAs in the patients’ samples revealed that whereas LOC285194 was significantly (*p* < 0.01) downregulated in patients with metastasis, lncRNAs HOTAIR and MALAT1 were even more significantly (*p* < 0.0001) elevated in patients with metastases. These results are presented in Figure 3.

### lncRNA Signature and Disease Progression

In addition to studying a role of lncRNA signature in determining liver metastases, we also evaluated if the lncRNA signature correlated with disease progression, as evidenced by staging of the primary colorectal cancer within 5 years of initial diagnosis. When we carefully evaluated the history of 17 patients (15 patients with liver metastases within 5 years of initial diagnosis vs. the five patients without any metastases within 5 years of initial diagnosis), we found a clear correlation of a positive signature (downregulated LOC285194, upregulated HOTAIR and MALAT1) with colorectal cancer progression (Table 2). More than 41% patients with a positive signature advanced to stage IV colorectal cancer. In the negative group (upregulated LOC285194, downregulated HOTAIR and MALAT1), a majority of patients still had a low grade tumor with only one out of five patients reporting advanced stage tumor.

**TABLE 2:** LncRNA signature and disease progression.

**Signature**	**Stage I/II**	**Stage III**	**Stage IV**
Positive (LM)	5/12 (41.67%)	2/12 (16.66%)	5/12 (41.67%)
Negative (NM)	4/5 (80%)	0/5 (0%)	1/5 (20%)

*Positive signature: Downregulated LOC285194, upregulated HOTAIR/MALAT1. Negative signature: Upregulated LOC285194, downregulated HOTAIR/MALAT1.*

## Discussion

Metastatic disease is often the primary cause of cancer-related deaths. Colorectal cancer is a major cancer worldwide and among several organs, liver is the primary metastatic site for metastatic colorectal cancers. Colorectal cancers can metastasize to several other organs as well (Riihimaki et al., 2016) and there is a debate if peritoneal carcinomatosis is the end stage of disease subsequent to hepatic metastases (Pretzsch et al., 2019). In view of the high rate of mortality associated with liver metastatic colorectal cancers, this research topic is an important one to be understood and elucidated. Further, as one of the novelty of our evaluation, we studied the expression levels of lncRNAs in liver metastatic colorectal cancers along with the establishment of a lncRNA signature that can potentially be linked to poor survival and outcome. LncRNAs belong to the class of non-coding RNAs that have long been considered junk RNAs in the human cells. However, slowly but surely their importance as disease biomarkers is being understood (Ahmad, 2016).

In this study, we evaluated several lncRNAs for their possible differential expression in the liver metastases, compared to the adjacent non-cancerous tissues. As expected, not all lncRNAs were significantly differentially expressed. H19 has been reported to differentially expressed in liver metastases of different cancers (Fellig et al., 2005). However, in our clinical samples, we did not find this lncRNA to be of relevance. ncRAN is another lncRNA whose downregulation has been reported in liver metastases (Qi et al., 2015). In our analysis, the lncRAN was significantly downregulated. Similarly, there is evidence for increased expression of lncRNA LINC01296 as a prognostic biomarker for colorectal cancers (Qiu and Yan, 2015) and our evaluation suggests only modest increased expression of this lncRNA in liver metastatic colorectal cancers. LncRNA RP11-462C24.1 has been reported to expressed at lower levels in metastatic colorectal cancers (Shi et al., 2014) but we did not find this lncRNA to be differentially expressed in our study. Similar observations were made for lncRNAs DANCR, ATB, lincRNA-ROR, SNHG20 and GAS5 all of which have also been shown to correlate with poor prognosis of colorectal cancers (Yin et al., 2014; Liu et al., 2015; Li et al., 2016b; Yue et al., 2016; Zhou et al., 2016) but we did not find them to be expressed at significantly different levels in metastases compared to the adjacent normal tissue. PCAT-1 has been suggested to be biomarker for poor prognosis of colorectal cancers (Ge et al., 2013) and we found the levels of this lncRNA only slightly elevated. Colorectal patients with high ZFAS1 levels have been reported to have shorter relapse-free survival and overall survival (Wang and Xing, 2016) and we found some relevance of this lncRNA to liver metastases. MEG3’s downregulation has been reported to associate with poor prognosis (Yin et al., 2015) but in our analysis the levels of this lncRNA were not significantly different. Thus, we tested a number of lncRNAs in our study, primarily based on the published literature but our results did not always corroborate the earlier published findings. While this could be attributed to many factors, including the sample size as well the geographical location and possible influence of race/ethnicity, only more detailed studies focused on elucidating such influences can provide a more definite answer.

In recent years there has been some interest in lncRNAs-based signatures in colorectal cancers. For example, a study identified eight lncRNAs associated with autophagy and associated lncRNA based signature with survival through their high vs. low risk groups stratifications (Wei et al., 2020). Then a three lncRNA-based signature was associated with prognosis potential in colorectal cancers (Liu et al., 2020) and a nine lncRNA signature was proposed to predict survival of colorectal patients (Zong et al., 2021). The process of epithelial-mesenchymal transition (EMT) is an important one connected to many aspects of tumorigenesis (Ginnebaugh et al., 2014) and EMT-regulating lncRNAs-based signatures can predict overall survival as well as disease-free survival of colorectal cancer patients (Liu et al., 2021). Similar to these EMT-regulating lncRNAs, even the signature comprising of stem cells-regulating RNAs (Wang et al., 2021) and immune-related lncRNAs (Qin et al., 2021) has been reported to be prognostic importance.

Our results are in agreement with a few earlier reports in the literature. For example, lncRNA HOTAIR has been reported to associate with liver metastases of colorectal cancers (Kogo et al., 2011). Similarly, MALAT1 is also upregulated in high grade colorectal cancers and its expression correlates with poor prognosis (Zheng et al., 2014). HOTAIR and MALAT1 are lncRNAs whose overexpression correlates with poor prognosis. On the other hand, it is the lower expression of lncRNA LOC285194 which correlates with liver metastases of colorectal cancers, as determined in this study. Incidentally, lower expression of LOC285194 has been related to poor prognosis in colorectal cancers (Qi et al., 2013). Despite some evidence for the role of HOTAIR, MALAT1 and LOC285194 individually as prognostic biomarkers, our study is the first to combine their power as a possible lncRNA signature for the prediction of liver metastases. Moreover, we also presented evidence supporting a connection of this lncRNA signature with disease progression which could result in poor overall survival. The next step would be to sue this knowledge to target these lncRNAs for therapy as well as further elucidate the mechanism of action of these lncRNAs which could yield further targets for therapy.

## Data Availability Statement

The original contributions presented in the study are included in the article/supplementary material, further inquiries can be directed to the corresponding author/s.

## Ethics Statement

The studies involving human participants were reviewed and approved by Ethics Committee at the China-Japan Friendship Hospital. The patients/participants provided their written informed consent to participate in this study.

## Author Contributions

FL, Z-MS, and S-YD conducted the experiments. FL, Z-MS, X-DW, and NP analyzed the results. J-RZ performed the statistical analyses. M-GZ conceptualized/supervised the project and obtained the funding. FL, Z-MS, and J-RZ drafted the manuscript. All authors read and approved the manuscript.

## Conflict of Interest

The authors declare that the research was conducted in the absence of any commercial or financial relationships that could be construed as a potential conflict of interest.
